# Antidepressant emergent mood switch in major depressive disorder: onset, clinical correlates and impact on suicidality

**DOI:** 10.1097/YIC.0000000000000479

**Published:** 2023-06-09

**Authors:** Paolo Olgiati, Alessandro Serretti

**Affiliations:** Department of Biomedical and Neuromotor Sciences, University of Bologna, Bologna, Italy

**Keywords:** bipolar spectrum, hypomanic switch, major depressive disorder, suicide attempt, suicide ideation

## Abstract

Antidepressant (AD)- emergent mood switch (AEMS) is a common complication of bipolar depression. This study aimed to investigate the prevalence and clinical correlates of subthreshold AEMS (i.e. not fulfilling DSM criteria for hypomanic episodes) in major depressive disorder (MDD) and, prognostically, its impact on AD treatment outcome and suicidality. The study involved 425 outpatients with MDD followed during the acute phase (12 weeks) and continuation (weeks 13–28) AD treatment. AEMS was assessed through the Altman Self-Rating Mania scale (ASRM ≥ 6). Several clinical features differentiated individuals with or without subthreshold AEMS (n = 204 vs. 221): negative self-perception [odds ratio (OR) 1.017–1.565]; panic disorder (OR 1.000–1.091); subthreshold hypomanic episodes (OR 1.466–13.352); childhood emotional abuse (OR 1.053–2.447); lifetime suicidal behaviour (OR 1.027–1.236); AD-related remission (χ^2^ = 22.903 *P* < 0.0001) and suicide ideation (χ^2^ = 16.701 *P* < 0.0001). In AEMS earlier onset showed a strong correlation with bipolar spectrum disorder (overall score: *P* = 0.0053; mixed depression: *P* = 0.0154; subthreshold hypomania: *P* = 0.0150) whereas late-onset was associated with more severe suicidal behaviour (*P* < 0.001). In conclusion, our results demonstrate that subthreshold mood switches occur frequently in unipolar depression during acute AD treatment as well as in continuation phase. Time of switch onset seems to have the greatest diagnostic and prognostic value.

## Introduction

It is a common experience for depressive patients to rapidly change their mood into hypo-/manic polarity during antidepressant (AD) treatment. This AD-emergent mood switch (AEMS) appears to be closely related to bipolar depression. In randomised trials conducted to investigate AD use in bipolar depression AEMS has involved 8% - 11% of patients during acute phase treatment and 14–21% during continuation phase (Leverich *et al*., 2006). In the STEP-BD study, more than 40% of bipolar patients showed AEMS within the first 12 weeks of starting an AD (Truman *et al*., 2007). A meta-analysis has estimated that AEMS is 2.77 times more frequent in bipolar II disorder compared to major depressive disorder (MDD) and that bipolar I disorder is 1.78 times more likely to develop AEMS than bipolar II disorder (Bond *et al*., 2008). Finally, mood stabilisers have been shown to exert a protective effect against AEMS in bipolar patients receiving long-term AD treatment (Liu *et al*., 2017). The prognostic role of AEMS in bipolar depression is essentially related to an increased risk for rapid cycling (Altshuler *et al*., 1995; Mattes, 2006; Thase, 2013) and, although less systematically investigated, to suicidality. In a large sample of bipolar I and II individuals analysed during a major depressive episode, transitions to manic or mixed episodes were associated with lifetime histories of suicide attempts along with other risk factors such as past depressive recurrence, rapid cycling and alcohol use disorder (Perlis *et al*., 2010). In MDD, the occurrence of AEMS has been associated with markers of underlying bipolarity such as family histories of bipolar disorders (Wada *et al*., 2006; Gill *et al*., 2020) and younger age at illness onset (Benazzi, 1997; Akechi *et al*., 2019) or with subsequent bipolar diagnoses (Dumlu *et al*., 2011). These findings suggest that MDD plus AEMS might belong to the bipolar spectrum, however, any definitive conclusion is hindered by the lack of a universally accepted definition of AEMS (Berk *et al*., 2010). The International Society for Bipolar Disorders (ISBD) task force has described treatment-emergent manic, hypomanic or mixed episodes occurring within 8 weeks after introducing an AD treatment (Tohen *et al*., 2009). After the introduction of DSM-5, this criterion would allow changing the diagnoses of patients initially classified as MDD cases into bipolar I or II disorder– provided that the manic or hypomanic episode persists at a full syndromic level beyond the physiological effect of AD treatment (Terao and Tanaka, 2014) – an issue for several bipolar subjects whose onset polarity is depressive (Baldessarini *et al*., 2014). Conversely, subthreshold AEMS, which does not include DSM criteria for hypomanic/manic episode, can have a variable prevalence in MDD (Bhogaraju, 2016), but its definition and time course remain unclear. Moreover, it is necessary to disentangle the prognostic role of subthreshold AEMS in unipolar depression, by analogy to other bipolar features that have been shown to increase the risk for suicide ideation (Olgiati *et al*., 2006) and behaviour (Amin-Esmaeili *et al*., 2018; Choi *et al*., 2019; Rihmer and Rihmer, 2019) as well as AD treatment resistance (Dudek *et al*., 2010; Jha *et al*., 2018). In this study, our primary goal was to investigate the clinical correlates of subthreshold AEMS (in particular, bipolar spectrum, suicide ideation/behaviour and AD treatment response) in a well-characterised sample of MDD outpatients. In addition, we aimed to test the diagnostic and prognostic impact of including time of onset and longitudinal course in AEMS definition.

## Methods

### Subjects

This study analysed participants in the Combining Medications to Enhance Depression Outcomes (CO-MED) trial which was originally carried out to compare the benefits of administering two ADs or one AD plus placebo in MDD (Rush *et al*., 2011). CO-MED eligibility criteria included age between 18 and 75 years old, a primary Diagnostic and Statistical Manual of Mental Disorders (DSM)-IV-based diagnosis of non-psychotic MDD, and a 17-item Hamilton Depression rating scale (HAM-D_17_) score of at least 16. In this study, two additional inclusion criteria were applied: (1) to have attended medical meetings on days 7 and 14, reporting regular use of prescribed AD drugs; (2) an Altman Self-Rating Mania scale (ASRM) score <6 (Altman *et al*., 1997) during baseline assessment. Conversely, any bipolar or psychotic disorder was reason for exclusion, along with the need for inpatient treatment.

The CO-MED trial was conducted according to the Principles of the Helsinki Declaration, and its protocol was approved by ethical committees at local recruitment sites. All subjects who met the selection criteria were included in the CO-MED trial after obtaining their written informed consent. This research group certifies that data collected from the CO-MED trial were exclusively used for scientific investigation and, before obtaining access to them, the objectives of our investigation were clearly reported in the request form (Olgiati and Serretti, 2022).

### Treatment and data collection

The CO-MED trial was characterised by a single-blind placebo-controlled design and three treatment arms: a) escitalopram (10–20 mg) plus placebo; b) bupropion SR (150–450 mg) plus escitalopram (10–20 mg); c) venlafaxine XR (75–225 mg) plus mirtazapine (15–45 mg). The trial included a short-term treatment phase (weeks 0–12) followed by a continuation phase (weeks 12–28) (Rush *et al.*, 2011).

Research data were collected by a variety of assessment tools as reported in our previous publications (Serretti *et al*., 2021; Olgiati and Serretti, 2022; Olgiati *et al*., 2022) and including: (1) a sociodemographic form to investigate age, gender, ethnic group, education and monthly income; (2) the Mini International Neuropsychiatric Interview (Sheehan *et al.*, 1998) to perform a retrospective evaluation of depressive disorder (chronic or recurrent course; number of depressive episodes and age at onset of first episode) and subthreshold hypomanic episodes; (3) a battery of scales for cross-sectional clinical assessment including the 30-item Inventory of Depressive Symptomatology-Clinician Rating (IDS-C_30_) (Corruble *et al*., 1999) and 16-item Quick Inventory of Depressive Symptomatology (QIDS-C_16_) (Rush *et al.*, 2003), the Concise Associated Symptoms Tracking (CAST) (Trivedi *et al*., 2011b) for irritability and Concise Health Risk Tracking-Self Report (CHRT)(Trivedi *et al*., 2011a) for suicide ideation (last 24 h), the ASRM (Altman *et al*., 1997) to ascertain hypomanic symptoms during depressive episode and the Work and Social Adjustment Scale (Mundt *et al.*, 2002) to determine functional impairment; (4) the Psychiatric Diagnostic Screening Questionnaire (Zimmerman and Mattia, 1999) to investigate comorbid mental disorders including panic disorder, generalised anxiety, obsessive-compulsive disorder, social phobia, post-traumatic stress disorder and alcohol and substance use disorders; and 5) two questionnaires which were specifically developed for CO-MED project to investigate lifetime suicidal behaviour and experiences of neglect and abuse during childhood (Serretti *et al*., 2021; Olgiati and Serretti, 2022).

### Antidepressant emergent mood switch, bipolar spectrum and prognostic variables

AEMS was defined by an ASRM score of 6 or higher, that is, considered to be a reliable cut-off score (sensitivity: >85; specificity: >87%) for hypomanic manifestations (Altman *et al*., 1997). Onset and longitudinal course of AEMS were defined using alternative criteria as follows: (1) AEMS-1: an ASRM score ≥6 reported at any time-point after baseline assessment. (2) AEMS-2: an ASRM score ≥6 reported once or more times after baseline but within 12 weeks of treatment. This time horizon is similar to those used in several studies that have investigated hypomanic or manic switch during acute AD treatment (Truman *et al*., 2007; Frye *et al*., 2009; Chen *et al*., 2022) and to 8-week limit proposed by the ISBD (Tohen *et al*., 2009). Hence, likely or possible subthreshold AEMS should be considered if hypomanic symptoms not meeting syndromal criteria emerge within 12 weeks of AD initiation (Gitlin, 2018). Conversely, the onset of a hypomanic switch beyond this time limit could reflect either a lower sensitivity to AD-induced mood activation (in fact, these subjects need a longer exposure to AD effects) or affective shifts not related to AD treatment. Therefore, we classified late switchers apart; (3) AEMS-3: both criteria were required: (I) ASRM < 6 until day 14; and (II) ASRM ≥ 6 during at least two consecutive visits after day 14. The first criterion was selected to reduce the risk for including spontaneous mood shifts, not related to AD treatment. The second criterion was used to warrant that all patients had a homogeneous course of AEMS. Patients who had ASMR≥6 episodes without fulfilling AEMS-3 criteria were classified as mood shift (MS) cases.

Bipolar spectrum variables included: (1) age at onset of first depressive episode (Benazzi, 2009); (2) mood disorder recurrence (number of episodes/illness years) (Mazzarini *et al*., 2018); (3) hypomanic symptoms occurring within major depressive episode (ASRM items): cheerfulness; self-confidence; reduced need for sleep; talkativeness; goal-oriented hyperactivity; (4) mixed depression (MxD), defined as a major depressive episode with three or more concurrent hypomanic symptoms (Akiskal *et al.*, 2005); (5) lifetime subthreshold hypomania: a period of elated or irritable mood with at least two concurrent hypomanic symptoms (MINI interview), which did not fulfill DSM criteria for hypomanic/manic episode (Angst *et al*., 2003; Serretti *et al.*, 2021). Besides analysing each bipolar spectrum variable an overall 0–5 bipolar score was calculated by adding age of onset <21 years (1 point), mixed depression (2 points) and subthreshold hypomania (2 points). A score of ≥3 was used as an index of high bipolar risk. Prognostic variables included: (1) AD response: >50% decrease in week 6 QIDS score from baseline score; (2) depression remission: week 6 QIDS ≤5; (3 suicidal ideation, assessed through the last three CHRT items: item 10: ‘I have been having thoughts of killing myself’; item 11: ‘I have thoughts about how I might kill myself’; item 12: ‘I have a plan to kill myself’. Suicide ideation was measured at baseline and after 7, 14, 28 and 42 days; d) lifetime most severe suicidal behaviour that was rated via a 0 (no suicidal tendency) to 7 (suicide attempt with definite intent to die) scale

### Comparisons and statistical analysis

Statistical analysis was conducted to investigate the distribution of sociodemographic features, depressive and hypomanic symptoms, psychiatric comorbidities, bipolar spectrum and prognostic variables using different AEMS definitions as previously explained. In the first set of comparisons, sample was subdivided into AEMS-1 and no AEMS groups. Subsequently, statistical analysis was replicated to compare individuals without mood activation (ASMR < 6) to early and late switchers according to AEMS-2 definition and, finally, to switching groups as defined by AEMS-3. A preliminary power analysis was conducted to estimate minimum detectable effect sizes (Cohen’s *d*) or percentual variations, considering a type I error (alpha level) of 0.05 and a type II error (1-power) of 0.20 (Abraham and Russell, 2008). Univariate analyses included Student’s *t*-test and ANOVA (plus Bonferroni post-hoc test) for continuous variables, whereas the Chi-square test was used for categorical variables. Mantel-Haenszel test for trend (MH chi-square) was preferred to simple Chi-square test to analyse ordinal variables (in our case, AEMS groups that were stratified over a continuum from individuals without any mood switch to early switchers (AEMS-2) or AEMS-3 cases). Significance threshold was set at alpha=0.05, without a formal correction for multiple testing given the exploratory nature of our analysis (Amrhein *et al*., 2019). Multivariate comparisons were performed by means of multiple logistic regression (MLR) analysis. Statistical software used to perform univariate and multivariate analyses was OpenStat version 8 December 2014 (https://openstat.info/OpenStatMain.htm). Power analysis and effect size calculation were performed by means of G*Power 3 program that was developed at Düsseldorf University (Germany) (Faul *et al.*, 2007).

## Results

### Sample’s characteristics and power analysis

Our original sample included 482 patients. Of them, 57 were not analysed because they had at least one missing visit during the first two treatment weeks or reported ASRM ≥ 6 during baseline assessment. Therefore, 425 patients were included in the current analysis. Their mean age was 43.42 ± 12.54 years; 125 patients (29%) were males and 291 (68%) were of Caucasian origin. Illness onset occurred at 23.27 ± 13.39 years of age, which implied an average illness length of 19.60 ± 13.79 years. The current episode overall depression score (IDS-C_30_) was 38.57 ± 9.29 at baseline and 20.55 ± 11.37 after 6 weeks of AD use (t = 25.30 *P* < 0.0001). Suicide ideation score decreased from 2.00 ± 2.54 at baseline to 1.09 ± 1.94 at week 6 (t = 6.32 *P* < 0.0001), but 177 patients (42%) had residual suicidal ideation (CHRT≥1). The average lifetime suicidal behaviour score was 2.11 ± 2.31: for 235 patients (55.2%) the worst suicidal behaviour was suicide ideation without any specific plan to kill themselves whereas 49 patients (10%) reported previous attempts with defined intent to die. Treatment combinations were as follows: escitalopram plus placebo: n = 147; bupropion plus escitalopram: n = 143; venlafaxine plus mirtazapine: n = 134.

AEMS-1 was reported in 204 patients (48%), without significant differences between treatment groups (escitalopram plus placebo: 70/147; bupropion plus escitalopram: 68/143; venlafaxine plus mirtazapine: 75/134 Chi-square: 0.031 d.f = 2, *P* = 0.984).

Power analysis suggested that our sample might have an adequate power (0.8) to detect small-medium effect sizes (d = 0.27) or differences of at least 7.5% in comparisons between with and without AEMS-1 groups.

### AEMS-1 vs. no AEMS

By applying AEMS-1 definition, comparison groups were similar regarding sociodemographic features (Table 1). In terms of clinical profile, subjects with AEMS-1 were characterised by higher scores of negative self-perception (*P* = 0.0467) and early awakening (*P* = 0.0328) than those without AEMS counterpart (Table 1). By performing MLR analysis, negative self-perception [odds ratio (OR) 1.017–1.565] and panic disorder (OR 1.000–1.091) emerged as independent predictors of AEMS-1. Conversely, interpersonal sensitivity (OR 0.647–0.960) and early awakening (OR 0.716–0.978) were protective factors (Table 1).

**Table 1:** AEMS-1: analysis of sociodemographic features, comorbid mental disorders and depressive symptom profile

	AEMS-1	No AEMS	
	(n = 204)	(n = 221)	*P*-value
Sociodemographic variables			
Age	43.47 ± 12.34	43.37 ± 12.75	0.9349
Sex (%M)	58 (0.28)	67 (0.30)	0.6699
Ethnicity (% Caucasian)	135 (0.66)	156 (0.70)	0.3287
Education (years)	13.70 ± 3.04	13.78 ± 3.16	0.7840
Work/social impairment	27.35 ± 8.46	26.67 ± 8.79	0.4187
Depression symptoms			
IDS-C30 (total score)	38.74 ± 9.05	38.42 ± 9.52	0.7237
Negative self-outlook	1.98 ± 0.96	1.80 ± 0.94	**0.0467**
Negative outlook for future	1.52 ± 0.89	1.52 ± 0.82	0.9492
Loss of pleasure	1.46 ± 0.93	1.52 ± 0.87	0.4617
Psychomotor agitation	0.72 ± 0.63	0.67 ± 0.61	0.4904
Psychomotor slowing	0.78 ± 0.64	0.69 ± 0.62	0.1181
Anxious mood	1.85 ± 0.78	1.79 ± 0.82	0.4354
Irritability (CAST)	0.75 ± 0.44	0.70 ± 0.56	0.2693
Interpersonal sensitivity	1.35 ± 1.04	1.52 ± 1.03	0.0792
Difficulty in falling asleep	1.82 ± 1.29	1.84 ± 1.29	0.8828
Middle nocturnal insomnia	2.07 ± 1.11	2.07 ± 1.14	0.9587
Early awakening	1.07 ± 1.25	1.33 ± 1.27	**0.0328**
Poor concentration	1.75 ± 0.81	1.72 ± 0.72	0.6819
Comorbid disorders			
Panic disorder (PD)	4.57 ± 4.76	3.73 ± 4.67	0.0669
Generalised anxiety (GAD)	6.65 ± 3.06	6.32 ± 3.34	0.2890
Obsessive-compulsive (OCD)	0.97 ± 1.66	0.93 ± 1.49	0.8018
Binge eating (BED)	2.52 ± 3.02	2.09 ± 2.99	0.1379
Social phobia (SP)	5.55 ± 5.10	5.03 ± 4.98	0.2908
Post-traumatic stress (PTSD)	3.62 ± 4.11	3.07 ± 3.94	0.1594
Alcohol use disorder (AUD)	0.66 ± 1.50	0.48 ± 1.20	0.1674
AEMS predictors	OR	(95% CI)	
Negative self-outlook	1.261	1.017–1.565	
Interpersonal sensitivity	0.788	0.647–0.960	
Early awakening	0.837	0.716–0.978	
Panic disorder	1.044	1.000–1.091	

Significant differences (*P* < 0.05) are in bold.

Multiple logistic regression (MLR) analysis was performed including all variables associated with *P* < 0.10 at univariate level. MLR model: Chi-square=17.468 d.f = 4, *P* = 0.0016.

AEMS-1: at least one ASMR≥6 episode after baseline assessment; CI, confidence interval; OR, odds ratio.

The distributions of hypomanic symptoms and bipolar spectrum markers are displayed in Table 2. Patients who developed AEMS-1 more often showed subthreshold hypomania (*P* = 0.0110), mixed depression (*P* = 0.0320), higher bipolar score (*P* = 0.0376), higher levels of self-confidence (*P* = 0.0108) and increased talkativeness (*P* = 0.0006) in comparison with no AEMS group. As regards prior traumatic experiences, AEMS-1 included more cases of childhood emotional abuse (*P* = 0.0220) (Table 2). By performing MLR analysis subthreshold hypomania was confirmed to be the strongest independent predictor of AEMS-1 (OR 1.466–13.352), followed by increased talkativeness (OR 1.094–2.145) and childhood emotional abuse (OR 1.053–2.447) (Table 2).

**Table 2:** AEMS-1 groups: bipolar spectrum variables and childhood traumas

	AEMS-1	No AEMS-1		
	(N = 204)	(N = 221)	*t* or *χ*^2^	*P*-value
Bipolar spectrum variables				
Age of onset	23.17 ± 13.01	33.36 ± 13.75	0.143	0.8865
Recurrence (episodes/year)	0.42 ± 0.92	0.31 ± 0.53	1.370	0.1631
Lifetime subthreshold hypomania	28 (0.137)	14 (0.063)	6.506	**0.0110**
Mixed depressive episode	24 (0.117)	13 (0.059)	4.618	**0.0320**
Overall bipolar score	1.04 ± 1.20	0.83 ± 0.88	2.061	**0.0377**
Intra-MDE hypomanic symptoms				
Cheerfulness	0.21 ± 0.45	0.11 ± 0.33	2.561	**0.0108**
Increased self-confidence	0.22 ± 0.52	0.13 ± 0.42	1.939	0.0513
Increased talkativeness	0.43 ± 0.82	0.19 ± 0.53	3.430	**0.0006**
Reduced need for sleep	0.37 ± 0.81	0.35 ± 0.87	0.240	0.8106
Hyperactivity	0.15 ± 0.54	0.09 ± 0.41	1.415	0.1538
Childhood traumas				
Neglect	89 (0.436)	80 (0.362)	2.444	0.1180
Emotional abuse	101 (0.495)	85 (0.384)	5.261	**0.0220**
Physical abuse	50 (0.245)	44 (0.199)	1.303	0.2540
Sexual abuse	52 (0.254)	52 (0.235)	0.221	0.6390
AEMS predictors	OR	(95% CI)		
Subthreshold hypomania	4.423	1.466–13.352		
Increased talkativeness	1.532	1.094–2.145		
Emotional abuse	1.605	1.053–2.447		

Significant differences (*P* < 0.05) are in bold. Percentages are in brackets.

Multiple logistic regression (MLR) analysis was performed including all variables associated with *P* < 0.10 at univariate level. Overall model fit: Chi-square=29.562 d.f = 7, *P* = 0.0001.

AEMS-1: at least one ASMR≥6 episode after baseline assessment; CI, confidence interval; OR, odds ratio.

Suicidal ideation was marginally lower in AEMS-1 patients compared to no AEMS group at baseline (*P* = 0.0561) and significantly lower in subsequent weeks (Table 3). Individuals who developed AEMS-1 were characterised by a better prognosis at week 6, being associated with more cases of response (χ^2^ = 24.192 *P* < 0.0001) and remission (χ^2^ = 22.903 *P* < 0.0001) and fewer cases of residual SI (χ^2^ = 16.701 *P* < 0.0001). By contrast, from a lifetime perspective, suicide behaviour was more severe in AEMS-1 group (*P* = 0.0140) (Table 3). In multivariate analysis, negative self-perception (OR 1.014–1.634) and lifetime suicidal behaviour (OR 1.027–1.236) were positively related to AEMS-1 whereas suicide ideation was inversely correlated (OR 0.804–0.958).

**Table 3:** AEMS-1 groups: analysis of suicidality and treatment outcome

	AEMS-1	No AEMS-1		
	(N = 204)	(N = 221)	*t* or *χ*^2^	*P*-value
Suicide ideation (CHRT 10–12)				
Baseline	1.76 ± 2.50	2.23 ± 2.56	1.916	0.0561
Week 1	0.95 ± 1.73	1.56 ± 2.10	3.206	**0.0016**
Week 2	0.86 ± 1.61	1.49 ± 2.06	3.333	**0.0011**
Week 4	0.68 ± 1.36	1.39 ± 1.96	4.130	**0.0001**
Week 6	0.72 ± 1.65	1.47 ± 2.12	3.758	**0.0002**
Lifetime suicidal behaviour				
Worst behaviour (score 0–7)	2.40 ± 2.47	1.85 ± 2.12	2.456	**0.0140**
Outcome variables (day 42)				
Response	108 (0.593)	62 (0.337)	24.192	**<0.0001**
Remission	75 (0.412)	34 (0.184)	22.903	**<0.0001**
Residual suicide ideation	38 (0.208)	76 (0.413)	16.701	**<0.0001**
AEMS predictors	OR	(95% CI)		
Lifetime suicidal behaviour	1.127	1.027–1.236		
Negative self-perception	1.287	1.014–1.634		
Suicide ideation	0.878	0.804–0.958		

Significant differences (*P* < 0.05) are in bold. Percentages are in brackets.

Available patients: n = 366.

Multiple logistic regression (MLR) analysis was performed to test the association between AEMS and lifetime suicidal behaviour by controlling for depression severity, suicide ideation, negative self-perception, and negative outlook on future and childhood traumas. Overall model fit: Chi-square=23.606 d.f = 9, *P* = 0.0050.

AEMS-1: at least one ASMR ≥ 6 episode after baseline assessment; CI, confidence interval; OR, odds ratio.

### Alternative AEMS-2 and AEMS-3 definitions

AEMS-2 definition was based on the time onset of the mood switch. Hence, 152 patients switched within week 12 (mean time to switch: 35.49 ± 23.83 days) and 52 later (mean time to switch: 139.67 ± 32.27 days). The number of visits with ASRM ≥ 6 was marginally higher in the earlier- compared to late-switching group (3.19 ± 2.32 vs. 2.53 ± 1.42 *P* = 0.0556)

We sought to ascertain whether bipolar spectrum and suicide-related variables followed an increasing trend from patients who never switched (N = 221) to early switchers, the most likely AEMS group. Late switchers were supposed to have intermediate characteristics. Overall, bipolar spectrum variables confirmed the expected trend: mixed depression (MHχ^2^, *P*  = 0.0154), subthreshold hypomania (MHχ^2^ *P* = 0.0150) and bipolar risk score ≥3 (MHχ^2^, *P* = 0.0053) were more represented in early switchers compared to individuals who did not switch; on the contrary, late switchers were not statistically different from no switch group (Table 4). Instead, as regards suicidality, late switchers were associated with more severe suicidal behaviour compared to other groups (F = 6.52, *P* < 0.001) (Table 4).

**Table 4:** AEMS-2: distribution of bipolar spectrum and suicide-related variables

AEMS-2: early switchers (individuals who develop their mood switch within week 12) may have true AEMS; in late switchers, mood changes are less likely to reflect AEMS				
	No switch	Late switchers	Early switchers	
	(a)	(b)	(c)	
Bipolar spectrum variables				
Subthreshold hypomania				**MH**χ^**2**^ ***P* = 0.0150**
No	207	45	131	**c vs. a: *P* < 0.0001** c vs. b: *P* = 0.9489 b vs. a: *P* = 0.0827
Yes	14	7	21	
Mixed depression				**MH**χ^**2**^ ***P* = 0.0154**
No	208	48	132	**c vs. a: *P* = 0.0150** c vs. b: *P* = 0.2910 b vs. a: *P* = 0.6269
Yes	13	4	20	
Bipolar risk score ≥3				**MH**χ^**2**^ ***P* = 0.0053**
No	204	46	126	**c vs. a: *P* = 0.0051** c vs. b: *P* = 0.3406 b vs. a: *P* = 0.3689
Yes	17	6	26	
Suicidality				
Lifetime suicide behaviour (severity: 0–7)	1.85 ± 2.12	3.12 ± 2.78	2.15 ± 2.32	**F = 6.5 *P* < 0.001**
				c vs. a: *P* = 0.1910 **c vs. b: *P* = 0.0160** **b vs. a: *P* < 0.0001**

Significant *P* values are in bold: *P* < 0.05 (univariate tests) and *P* < 0.0167 after Bonferroni’s correction (multiple testing).

MH: Mantel-Haenszel test for trend F: ANOVA.

AEMS-3 definition considered the longitudinal course of AEMS: 88 patients had two or more consecutive visits with ASRM ≥ 6 after day 14 (AEMS-3) whilst 116 experienced mood shifts without AEMS-3. The number of visits with ASRM ≥ 6 was 4.40 ± 1.94 in the former group compared to 1.97 ± 1.64 reported in the latter group (*P* < 0.0001). Bipolar spectrum variables did not follow an increasing trend from no AEMS to AEMS-3 group as expected: subthreshold hypomania was more often reported in patients who experienced mood switch (MHχ^2^, *P P* = 0.0223), but without statistically significant distinction between individuals with or without AEMS-3 (Table 5). Mixed depression (MHχ^2^, *P P* = 0.2249) and bipolar risk score ≥3 (MHχ^2^, *P P* = 0.073) were similarly distributed between all comparison groups (Table 5). Conversely, patients with AEMS-3 were characterised by more severe suicidal behaviour in comparison with other groups (F = 4.11, *P P* = 0.020).

**Table 5:** AEMS-3: distribution of bipolar spectrum and suicide-related variables

AEMS-3: possible AEMS is defined by both criteria (1) and (2) as follows: (1) no mood switch (ASRM ≥ 6) until day 14 and (2) after day 14, a switch is reported during at least two consecutive visits. Switchers who do not fulfill AEMS-3 criteria are classified as mood shift (MS) cases				
	No switch	Mood shift	AEMS-3	
	(a)	(b)	(c)	
Bipolar spectrum variables				
Subthreshold hypomania				**MH**χ^**2**^ ***P* = 0.0223**
No	207	99	76	c vs. a: *P* = 0.0369 c vs. b: *P* = 0.9489 b vs. a: *P* = 0.0208
Yes	14	16	12	
Mixed depression				MHχ^2^ *P* = 0.2249
No	207	100	80	c vs. a: *P* = 0.3952 c vs. b: *P* = 0.3019 b vs. a: *P* = 0.0223
Yes	14	16	8	
Bipolar RISK score ≥3				MHχ^2^ *P* = 0.0734
No	203	96	76	c vs. a: *P* = 0.1411 c vs. b: *P* = 0.5711 b vs. a: *P* = 0.0199
Yes	18	19	12	
Suicidality				
Lifetime suicide behaviour (severity: 0–7)	1.80 ± 2.09	2.30 ± 2.40	2.57 ± 2.57	**F = 4.1 *P* = 0.020**
				**c vs. a: *P* = 0.0070** c vs. b: *P* = 0.4440 b vs. a: *P* = 0.0520

Significant *P* values are in bold: *P* < 0.05 (univariate tests) and *P* < 0.0167 after Bonferroni’s correction (multiple testing).

MH: Mantel-Haenszel test for trend; F: ANOVA.

## Discussion

This study investigated mood switches in unipolar depression, reporting a number of significant clinical correlates. In line with prior literature, which emphasised the connection between mood switch and bipolar liability (Benazzi, 1997; Wada *et al*., 2006, 2013; Dumlu *et al.*, 2011; Barbuti *et al*., 2017), two bipolar spectrum variables such as subthreshold hypomania (Angst *et al*., 2003; 2010) and mixed depression (Akiskal *et al*., 2005; Benazzi and Akiskal, 2005; Brancati *et al*., 2019) were associated with mood switch. Other switch correlates, such as panic disorder, attempted suicide and childhood emotional abuse, although not strictly classifiable as bipolar spectrum markers, could nevertheless mirror a bipolar diathesis. In this sense, we would mention studies that display a correlation between panic disorder and bipolar illness (Zimmermann *et al*., 2009; Miola *et al*., 2023) and those supporting the role of underlying bipolarity in depression-related suicide, reviewed by Rihmer and Rihmer (2019). Furthermore, a meta-analysis has suggested that traumatic experiences occurring in childhood might increase the likelihood of developing bipolar disorder by 2.63 folds and emotional abuse by over four folds (Palmier-Claus *et al*., 2016). On the other hand, variables that have been repeatedly associated with bipolar illness such as the age of onset (Benazzi, 2009; Jo *et al*., 2022; Miola *et al*., 2023) and higher recurrence of depressive episodes (Mazzarini *et al*., 2018), were not associated with mood switch in our sample, unlike observed in prior studies (Benazzi, 1997; Niitsu *et al*., 2015; Akechi *et al*., 2019). Notably, in terms of prevalence, nearly 50% of patients developed any mood switch during a 28-week follow-up, although true markers of bipolar liability such as subthreshold hypomania or mixed depression were reported in no more than one-tenth of the sample. This consideration suggests that an ASRM score ≥6 reported at any time point after baseline might not be enough specific as a definition of AEMS. In other terms, we could have gathered different types of mood switches under the same diagnostic category. Therefore, to select a more homogeneous sample, we developed alternative AEMS definitions based on onset and longitudinal course. Time of onset after the introduction of AD treatment has already been identified as a critical factor for the study of AEMS (Bhogaraju, 2016; Gitlin, 2018). Although empirical evidence is lacking, it has (arbitrarily) been proposed that an AEMS would be the emergence of a syndromal mania/hypomania within 8 weeks of either the initiation of the AD or an increase in its dose (Tohen *et al*., 2009). Our results extend the suitability of such a time window to subthreshold AEMS. In fact, individuals who experience a mood switch within 12 weeks from starting AD treatment appear to be at increased risk for bipolar spectrum disorder compared to other groups, including late switchers. Having a greater bipolar liability, these subjects could be more sensitive to the excitatory effects of AD medications, thereby developing hypomanic symptoms. The lag phase of AEMS is relatively short, averaging 35 days. Regarding the persistence of hypomanic symptoms, our data indicate that they could be detected during at least two consecutive visits (minimum period: 2 weeks) in about 40% of switchers (88/204). Using narrower criteria has reduced the prevalence of AEMS: whilst a broad definition such as AEMS-1 involved approximately half the sample, limiting AEMS to the first 12 weeks of treatment led to 35% of positive cases (152/425). In the end, after introducing a lag phase of 2 weeks and hypomanic manifestations for at least two consecutive visits as identification criteria, the prevalence of AEMS became 20% (88/425). To make a comparison, in previous studies AEMS prevalence ranged from 0.5 to 17% (Barbuti *et al*., 2017; Akechi *et al*., 2019). In a meta-analysis involving 51 studies and more than 95 000 patients with MDD who received AD medications, researchers estimated an 8.18% overall risk for mood-switching, corresponding to 3.42% per treatment year (Baldessarini *et al*., 2013). Our higher prevalence could reflect a different assessment procedure and diagnostic threshold for mood elevation. Elsewhere the most common approach has been to evaluate the risk of developing DSM-based hypomanic or manic episodes (Akechi *et al*., 2019; Barbuti *et al*., 2017; Wada *et al*., 2006, 2013). Here, conversely, we analysed subthreshold AEMS (i.e. not fulfilling DSM criteria for hypo-/manic episode) as defined by a self-report scale and explored mild and moderate hypomanic symptomatology. How can we explain AEMS in biological terms? We speculate that mood switch would originate from an imbalance of monoamine systems (mainly dopamine networks) that can also occur spontaneously but is aggravated by AD use (Tang *et al*., 2022). This idea is based on the dopamine hypothesis that has been developed to explain the alternance of manic and depressive episodes in bipolar disorders (Ashok *et al*., 2017). An elevation in striatal D2/3 receptor availability would lead to altered reward processing and the development of mania, which is followed by a compensatory increase in dopamine transporter levels to reduce dopaminergic neurotransmission. However, if, over time, D2/3 receptor levels reduce but dopamine transporter levels do not normalise, this would then lead to reduced dopaminergic transmission, leading to depression and, in turn, a compensatory upregulation of D2/3 receptor levels, precipitating a further phase switch (Ashok *et al*., 2017). AD drugs could interfere with this cycle not only by blockading monoamine transporters but also because they might induce changes in dopamine receptor sensitivity (Demontis *et al*., 2017). In bipolar spectrum depression AEMS could be generated by a similar mechanism, however patients who express more bipolar markers would need a shorter time exposure to AD treatment before switching to a state of mood activation. It must be noted that affective instability is not only a characteristic of bipolar disorders (De Prisco *et al*., 2022; Miola *et al*., 2022) but it is present in several other conditions – for example, borderline personality disorder (Chapman, 2019); PTSD (Dolan *et al*., 2023); ADHD; eating disorders (Prefit *et al*., 2019; Bodell *et al*., 2022; Leppanen *et al*., 2022) – as an expression of emotion dysregulation. In the CO-MED sample, psychopathological manifestations such as irritability, PTSD symptoms and binge eating are particularly frequent (Serretti *et al*., 2021; Olgiati and Serretti, 2022; Olgiati *et al*., 2022), so it might not be simple to differentiate true AEMS from affective changes that are related to emotion dysregulation. In fact, previous studies have shown that rapid changes from opposite mood states can occur in both affective and emotion dysregulation disorders, with substantial similarities regarding their expression and magnitude (Houben *et al*., 2016). Our findings suggest that time of onset might help in identifying the aetiology of mood switch, meaning that late-onset (>12 weeks) switch, being associated with lower bipolar spectrum risk, would more likely be triggered by emotion dysregulation. On the other hand, as an issue to understanding the switching phenomenon, cumulating evidence suggests that there might be anamnestic, neurobiological and clinical overlaps between emotion dysregulation and bipolar illness. In both conditions traumatic experiences occurring in childhood are common antecedents (Carvalho Fernando *et al*., 2014; Palmier-Claus *et al*., 2016): in our sample childhood emotional abuse, which has been found to correlate with mood switch and bipolar spectrum indicators (Olgiati and Serretti, 2022), could mediate the connection between emotion dysregulation and underlying bipolarity. Among patients with MDD, an excess of borderline personality diagnoses has been observed in those who develop AEMS (Barbuti *et al*., 2017) whereas a recent study has shown that bipolar spectrum symptomatology can fluctuate with emotion variability and reactivity (Sperry and Kwapil, 2022). According to neurophysiological research, the patterns of brain signal variability associated with greater emotional dysregulation would be located in the front-limbic-system (Kebets *et al*., 2021). The suggested mechanism involves hyper-activation of limbic regions responsible for emotion generation – in particular, the amygdala, hippocampus, and ventral striatum-, coupled with hypo-activation of the prefrontal cortex, which is responsible for cognitive control. This circuit has shown similar structural abnormalities in emotion dysregulation disorders – borderline personality disorder (Ruocco and Carcone, 2016; Schulze *et al*., 2016) and ADHD (Hoogman *et al*., 2017) – and bipolar disorders (Phillips and Swartz, 2014; Hibar *et al*., 2016).

The occurrence of hypomanic symptoms in MDD has often been characterised by a negative prognostic impact. For example, in a retrospective analysis of 1051 outpatients with DSM-IV MDD, higher scores on hypomanic symptoms scales (MDQ; HCL-32) were found to predict AD treatment resistance (Dudek *et al.*, 2010). Similarly, in a prior analysis of the CO-MED sample, baseline hypomanic symptoms were associated with lower remission rates at week 12 (Jha *et al*., 2018). In our study, AEMS was shown to exert contrasting short and long-term effects. During acute phase AD treatment AEMS emerged as a positive outcome predictor that was associated with AD response and a favourable course of suicide ideation. In the long-term, conversely, AEMS was a possible risk factor for suicide behaviour. These results were in line with a large naturalistic study that analysed 6105 patients with mood disorders: in the major depression subsample (n = 5117) manic/hypomanic symptoms, as measured by the AMSR, were inversely related to suicide ideation outcome whereas heightened suicide behaviour risk (Fiedorowicz *et al*., 2021).

Our last hypothesis was that AEMS could reflect an acceleration of AD response. In accordance with this view, a significant decline in suicide ideation was noted as early as the first treatment week in patients who later developed AEMS. Similarly, a previous study by Akechi *et al*. (2019) reported that early response (within week 3) to AD treatment increased the likelihood of mood switch.

Our study had important strengths. The CO-MED trail was characterized by a well-implemented design and a thorough clinical assessment and the sample was adequately powered to detect even small differences between comparison groups. Despite this, we must acknowledge some limitations of post-hoc analysis. A set of caveats involved the assessment of bipolar markers. Firstly, the ASRM scale did not explore hypomanic manifestations such as racing thoughts, distractibility, and excessive involvement in risky activities and behaviours, which are often observed in mixed depression (Perugi *et al*., 2015; Brancati *et al*., 2019). This did not allow us to ascertain the prevalence of mixed syndromes at baseline and within AEMS. Second, family history of bipolar illness – one of the most useful variables to distinguish bipolar II depression from MDD (Zimmerman *et al.*, 2013) – was not available for our sample. Such missing information could have hindered the identification of bipolar spectrum cases in AEMS group. Third, it was difficult to assess the impact of AEMS and bipolar markers on suicidality. In fact, no information was available about the characteristics of suicide attempts (impulsive or based on plans; use of potentially lethal methods; presence of intent to die, etc.) and symptomatology prior to their occurrence (suicidal ideation; hypomanic symptoms, etc.). Moreover, no scales were administered to explore psychological correlates of suicidal behaviour such as hopelessness (Sokero *et al*., 2003; Ribeiro *et al.*, 2018), thwarted belongingness and burdensomeness (Chu *et al.*, 2017). Finally, a main confounding diagnosis, not analysed by the CO-MED study, was borderline personality disorder. This condition is similar to MDD with AEMS in many aspects such as rapid changes in mood, negative self-perception, suicide attempts and emotional abuse experiences.

In conclusion, mood switch is a common phenomenon in MDD that involves patients with a clearcut bipolar diathesis but also those in whom bipolar spectrum markers are lacking. Many correlates of AEMS (childhood emotional abuse; suicide attempt; negative self-perception) are shared by subjects with bipolar disorder and different diagnostic entities such as borderline personality disorder (Durdurak *et al*., 2022) and might reflect their reduced capability to regulate emotions. Time of onset might be a useful parameter to consider in order to disentangle whether a rapid mood activation in a depressed patient is related to an underlying bipolarity that is worsened by AD treatment (AEMS) or, instead, it is an expression of emotional dysregulation. Further research is needed to elucidate the biological underpinnings of AEMS which could facilitate the development of preventive strategies.

## Acknowledgements

We thank the NIMH for having the possibility of analysing their data on the CO-MED sample. We also thank the authors of previous publications in this dataset, and foremost, we thank the patients and their families who accepted to be enrolled in the study. Data and biomaterials were obtained from the limited access datasets distributed by the NIH. The ClinicalTrials.gov identifier is NCT00590863.

The CO-MED trial was conducted according to the Principles of Helsinki Declaration and its protocol was approved by ethical committees at local recruitment sites. All subjects who met the selection criteria were included in the CO-MED trial after obtaining their written informed consent. This research group certifies that data collected from the CO-MED trial were exclusively used for scientific investigation and, before obtaining access to them, the objectives of our investigation were clearly reported in the request form (Olgiati and Serretti, 2022).

### Conflicts of interest

There are no conflicts of interest.
